# Horizontal Semicircular Canal Benign Paroxysmal Positional Vertigo Presenting With Biphasic Nystagmus in the Healthy Ear-Down Position

**DOI:** 10.7759/cureus.115178

**Published:** 2026-08-25

**Authors:** Akihide Ichimura

**Affiliations:** 1 Otolaryngology - Neurotology/Vestibular Disorders, Ichimura ENT Clinic, Tokyo, JPN

**Keywords:** benign paroxysmal positional vertigo, biphasic nystagmus, canalolithiasis, cupulolithiasis, horizontal semicircular canal

## Abstract

In horizontal semicircular canal benign paroxysmal positional vertigo (HSC-BPPV) due to canalolithiasis, biphasic nystagmus is typically observed in the affected ear down or in both ear-down positions during the supine head-roll test. This report describes a case of HSC-BPPV with biphasic nystagmus observed only in the healthy ear-down position.

A 59-year-old male had a one-day history of rotational vertigo when turning to the right. The supine positional nystagmus test produced left-beating horizontal nystagmus in the face-up position. In the left-ear-down position, there was biphasic nystagmus (i.e., left-beating horizontal nystagmus followed by right-beating horizontal nystagmus). In the right-ear-down position, there was right-beating horizontal nystagmus. The first-phase nystagmus observed in the left-ear-down position was stronger than the right-beating nystagmus in the right-ear-down position. The patient was initially diagnosed with left-sided HSC-BPPV (canalolithiasis), and the Lempert roll maneuver was performed. Re-examination the following day revealed findings consistent with right-sided HSC-BPPV (cupulolithiasis).

Biphasic nystagmus can occur with the presumed healthy ear-down during the supine head-roll test. Misidentification of the affected side in HSC-BPPV (canalolithiasis) may lead to an inappropriate therapeutic maneuver and suboptimal treatment. In the present case, the affected side was initially misidentified, and persistent direction-changing apogeotropic nystagmus consistent with HSC-BPPV (cupulolithiasis) was observed after the maneuver. Although the mechanism underlying this change remains speculative and is based on a single case, clinicians should recognize that biphasic nystagmus during the head-roll test does not necessarily identify the affected ear. Careful interpretation of all positional nystagmus findings is warranted before performing a therapeutic maneuver.

## Introduction

Benign paroxysmal positional vertigo (BPPV) is one of the most common peripheral vestibular disorders, caused either by otoconia moving freely within a semicircular canal (canalolithiasis) or by otoconia adhering to the cupula (cupulolithiasis). In canalolithiasis, changes in head position can cause otoconia to move within the semicircular canal, which generates endolymphatic flow that excites or inhibits the canal and induces nystagmus. Maintaining the head position helps stabilize the otoconia, and the nystagmus subsides thereafter. However, in some cases, the direction of nystagmus can reverse even without changes in head position [1]. Several hypotheses have been proposed to explain the mechanisms underlying biphasic nystagmus, but a consensus has not been reached. In horizontal semicircular canal (HSC)-BPPV (canalolithiasis), biphasic nystagmus is typically observed during the supine head-roll test, either in the affected-ear-down position or in both positions [2-8]. Here, we report an unusual case in which biphasic nystagmus was observed exclusively with the healthy ear in the dependent position during the supine head-roll test. Awareness of this atypical pattern is important because misidentification of the affected ear may lead to an inappropriate repositioning maneuver and unsuccessful treatment. We describe the clinical findings, discuss possible mechanisms underlying the observed nystagmus, and highlight the implications for routine neurotological practice.

## Case presentation

A 59-year-old man experienced rotational vertigo triggered by turning to the right in bed upon awakening and presented one day after symptom onset. The vertigo was not associated with hearing loss, tinnitus, headache, or other neurological symptoms. His medical history included hyperlipidemia and hyperuricemia, both managed with appropriate medications. There was no history of head trauma, surgery, or relevant familial disease.

Neurological examination showed no evidence of dysdiadochokinesis, dysmetria, tremor, or gait ataxia. No spontaneous or gaze-evoked nystagmus was observed. Ocular motor testing (i.e., smooth pursuit, saccades, and optokinetic nystagmus using a rotating drum) was unremarkable. The supine positional nystagmus test, bow test, and Dix-Hallpike test were performed and recorded using an infrared charge-coupled device camera. The duration of nystagmus, latency between the first and second phases of nystagmus, and maximum slow-phase velocity (mSPV) were quantified using two-dimensional video-oculography and the ImageJ software (National Institutes of Health, Bethesda, MD, USA) on a Windows-based system [9].

**Figure 1:**
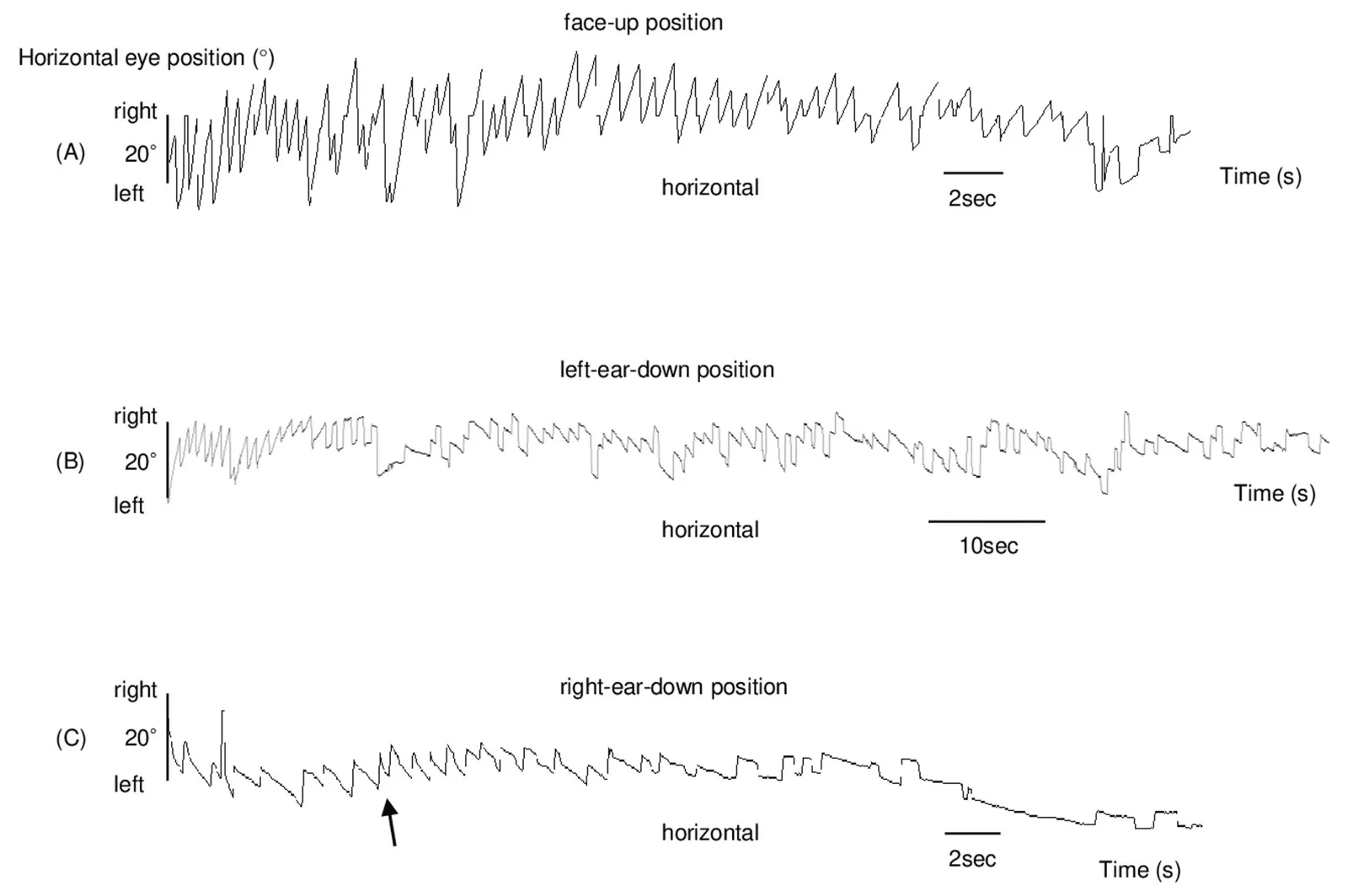
Video-oculographic recordings during the supine positional nystagmus test (A) Face-up position: left-beating horizontal nystagmus (maximum slow-phase velocity (mSPV), 75.2°/s; duration, 33 s); (B) Left-ear-down position: initial left-beating horizontal nystagmus (mSPV, 18.8°/s; duration, 16 s), followed by a nystagmus-free interval of 8 s and subsequent right-beating horizontal nystagmus (mSPV, 3.5°/s; duration, 64 s); (C) Right-ear-down position: right-beating horizontal nystagmus (mSPV, 11.2°/s; duration, 22 s). The arrow indicates the point at which the nystagmus suddenly increased in intensity.

The supine positional nystagmus test was performed with the patient seated on an electric treatment chair, which was tilted from a sitting to a supine position over 11 seconds. The patient was sequentially placed in the face-up, left-ear-down, and right-ear-down positions, then face-up positions. In the initial supine face-up position, left-beating horizontal nystagmus lasted 33 s. In the left-ear-down position, left-beating horizontal nystagmus lasted 16 s. After an 8-s latency without any change in head position, right-beating horizontal nystagmus persisted for 64 s. In the right-ear-down position, right-beating horizontal nystagmus lasted 22 s. The first-phase nystagmus in the left-ear-down position was stronger than the right-beating nystagmus in the right-ear-down position (Figure 1, Video 1). No bowing nystagmus was observed (Video 2). The Dix-Hallpike test elicited geotropic, direction-changing horizontal nystagmus in both head-hanging positions (Video 2), but biphasic nystagmus was not observed.

**Video 1:** The supine positional nystagmus test (first visit) Written informed consent to include this video in an open-access article was obtained from the patient.

**Video 2:** The bow test and Dix-Hallpike test (first visit) Written informed consent to include this video in an open-access article was obtained from the patient.

The presence of left-beating nystagmus in the supine face-up position suggested that the right ear was the affected side. However, the stronger nystagmus in the left-ear-down position compared to the right-ear-down position, as well as the biphasic nystagmus in the left-ear-down position, suggested that the left ear was the affected side. Despite these inconsistent findings, the patient was diagnosed with left-sided HSC-BPPV (canalolithiasis) based on the notable findings in the left-ear-down position [10]. A left Lempert 360° roll maneuver was subsequently performed [11]. The patient returned for follow-up the next day, reporting persistent rotational vertigo while supine and continued unsteadiness during the day. The supine positional nystagmus test revealed right-beating horizontal nystagmus lasting more than 78 s in the face-up position. Direction-changing apogeotropic nystagmus was observed in both ear-down positions, with stronger nystagmus in the left-ear-down position. The nystagmus disappeared after the head was rotated about 10° to the right from the face-up position (Video 3). Based on these findings, the patient was diagnosed with right-sided HSC-BPPV (cupulolithiasis) [10], and the Zuma maneuver was performed [12]. Upon follow-up three days later, the vertigo and nystagmus had both completely resolved. Table 1 summarizes the mSPV values and duration of nystagmus obtained during the supine positional test. 

**Video 3:** The supine positional nystagmus test (follow-up) Written informed consent to include this video in an open-access article was obtained from the patient.

**Table 1:** Characteristics of nystagmus observed during the supine positional nystagmus test mSPV: maximum slow phase velocity; °/s: degrees per second

	First-phase			Second-phase		
Visit	Position	Direction	mSPV, °/s	Duration, s	Direction	mSPV, °/s	Duration, s
First visit	Face-up	Leftward	75.2	33	‐	‐	‐
	Left-ear-down	Leftward	18.8	16	Rightward	3.5	64
	Right-ear-down	Rightward	11.2	22	‐	‐	‐
Follow-up	Face-up	Rightward	150.2	>78	‐	‐	‐
	Left-ear-down	Rightward	298.3	>6	‐	‐	‐
	Right-ear-down	Leftward	29.4	>15	‐	‐	‐

Written informed consent to publish anonymized clinical and video data was obtained from the patient. All reasonable efforts were made to protect patient identity.

## Discussion

This case highlights two important clinical considerations in HSC-BPPV (canalolithiasis). First, biphasic nystagmus can occur during the supine head-roll test not only in the affected-ear-down position but also in the healthy ear-down position. Second, if the Lempert roll maneuver is performed after incorrect identification of the affected side, canalolithiasis may convert to cupulolithiasis.

Typically, in previous reports, biphasic nystagmus in HSC-BPPV (canalolithiasis) has been observed either only when the affected ear is down or on both sides during the supine head-roll test [2-8]. However, biphasic nystagmus appeared only when the presumed healthy ear was down in the present case. This conclusion is supported by two findings: left-beating nystagmus was present in the supine face-up position during the initial examination; and the findings converted to right-ear HSC-BPPV (cupulolithiasis) after the left-sided Lempert 360° roll maneuver.

During the head-roll test in HSC-BPPV (canalolithiasis), nystagmus is typically stronger when the affected ear is down, compared to when the healthy ear is down [1]. Conversely, the bow and lean test can more accurately identify the affected side [13]. In the present case, left-beating nystagmus was observed in the supine face-up position, suggesting that the right ear was affected. However, stronger nystagmus was observed in the left-ear-down position versus the other side, and biphasic nystagmus was also present in the left-ear-down position. Based on these findings, the initial diagnosis was left-sided HSC-BPPV (canalolithiasis), and the Lempert roll maneuver was performed accordingly. However, there was subsequent conversion to right-sided cupulolithiasis the following day, indicating that the right ear was the affected side.

**Figure 2:**
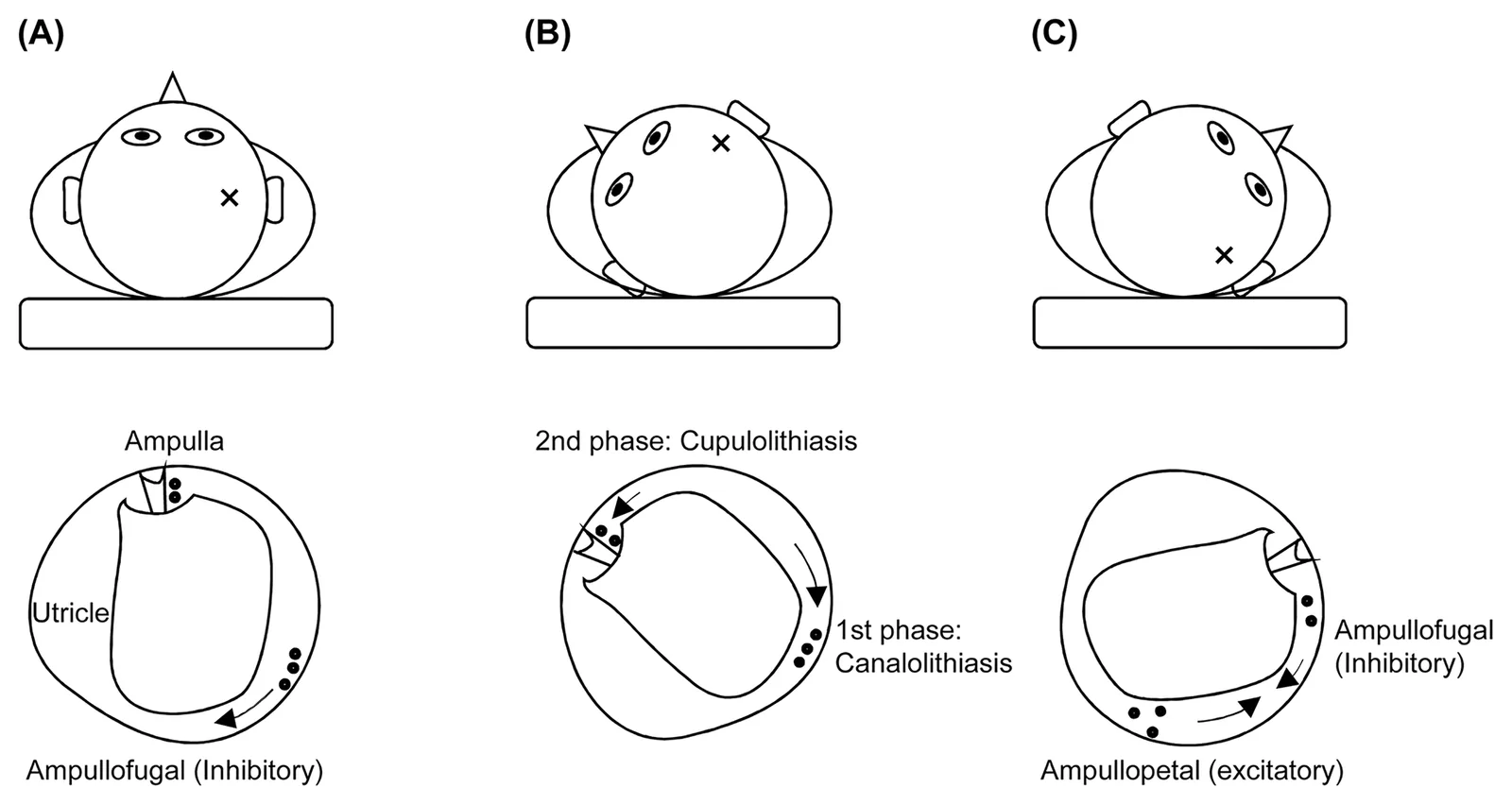
Proposed mechanism for the biphasic and asymmetric nystagmus responses (A) Face-up position: as the head is positioned supine, free-floating otoconia in the right horizontal canal shift toward the posterior part of the canal, generating an ampullofugal (inhibitory) response. (B) Left-ear-down position: free-floating otoconia in the posterior part move toward the utricle, generating an ampullofugal (inhibitory) response resulting in first-phase nystagmus. This inhibitory response is partially offset by an excitatory ampullopetal deflection of the cupula caused by cupulolithiasis. As the free-floating otoconia come to rest, the excitatory response generated by cupulolithiasis produces second-phase nystagmus. (C) Right-ear-down position: free-floating otoconia in the posterior part move toward the cupula and generate an ampullopetal (excitatory) stimulus. Simultaneously, otoconia attached to the cupula detach and migrate toward the utricle, producing an opposing ampullofugal (inhibitory) influence that counteracts the excitatory ampullopetal deflection of the cupula. As a result, the nystagmus is attenuated. Black-filled circles represent otoconia. Arrows indicate the direction of otoconial movement; × denotes the right (affected) ear. The relative strength of excitatory and inhibitory influences differs between the two positions, which explains the stronger net nystagmus in the healthy-ear-down position. Image credit: This figure was created by the author using Microsoft PowerPoint (Microsoft Corporation, Redmond, Washington, United States).

The findings may be explained by the coexistence of both canalolithiasis and cupulolithiasis in the right HSC (Figure 2) [6,7]. The biphasic nystagmus likely resulted from this, whereas its absence in the right-ear-down position can be attributed to the detachment of otoconia from the cupula [14]. In the face-up position, free-floating otoconia in the right canal likely moved toward the posterior nonampullary part and its utricle, producing ampullofugal inhibitory flow and left-beating nystagmus. Notably, because the head was repositioned to the left-ear-down position approximately 4 s after disappearance of the left-beating nystagmus, a second-phase right-beating nystagmus was not observed. However, this may have appeared if the examination was continued in the supine face-up position. In the left-ear-down position, otoconia in the posterior canal probably moved toward the utricle, producing the first-phase nystagmus, followed by the second-phase induced by cupulolithiasis [15]. Previous studies have reported that biphasic nystagmus tends to occur when the mSPV of the first phase exceeds 50°/s [3,4,6]. In our case, despite the relatively low mSPV of the first phase (18.8°/s), biphasic nystagmus may appear if there is coexisting canalolithiasis and cupulolithiasis [7].

The absence of biphasic nystagmus in the right-ear-down position may be explained by the stronger ampullopetal flow produced by otoconia moving toward the cupula, which overrides the ampullofugal flow of the detached otoconia. Moreover, the nystagmus intensified approximately 8 s after being placed in the right-ear-down position (Figure 1C, arrow), suggesting that the otoconia detached from the cupula and subsequently stopped moving toward the utricle. This assumption is further supported by the absence of biphasic nystagmus during the Dix-Hallpike test performed after the supine positional nystagmus test. During the supine positional nystagmus test, nystagmus was weaker in the right-ear-down (affected) position than in the left-ear-down (healthy) position, which is paradoxical based on Ewald’s second law [16]. In the right-ear-down position, otoconia in the posterior canal would normally move toward the cupula and generate an ampullopetal (excitatory) response. However, this excitatory response was likely reduced because otoconia detached from the cupula soon after the positional change and migrated toward the utricle, producing a competing ampullofugal (inhibitory) influence. Conversely, in the left-ear-down position, free-floating otoconia in the posterior part of the canal moved toward the utricle and produced an ampullofugal (inhibitory) response, which was partially offset by an excitatory ampullopetal deflection of the cupula caused by cupulolithiasis. Although there were opposing forces in both positions, nystagmus generated by cupulolithiasis is reportedly only one-third as strong as that generated by canalolithiasis [17]. In this case, the cancellation of the strong excitatory canal response in the right-ear-down position was more substantial than the cancellation of the inhibitory response in the left-ear-down position, resulting in a stronger nystagmus in the healthy ear-down position (Figure 2).

There are several proposed mechanisms for biphasic nystagmus in the affected-ear-down position: reversal of endolymphatic flow [2,8]; peripheral sensory adaptation [6]; and short-term adaptation of the vestibulo-ocular reflex (VOR) [3-5]. However, these mechanisms cannot fully explain the findings in our case. The first hypothesis, reversal of endolymphatic flow, assumes that second-phase nystagmus appears when the otoconia, which are driven by inertia, reverse direction after passing the lowest point. According to the Basset-Boussinesq-Oseen equation, which describes the motion of small particles in a viscous fluid, otoconia can reach terminal velocity almost instantaneously when settling through the endolymph [18]. Fluid dynamic models of BPPV have also shown that inertia only plays a small role in otoconial movement [17]. Therefore, this mechanism cannot account for the prolonged second-phase nystagmus (>60 s) observed in the present case [3,19]. The second hypothesis, peripheral sensory adaptation, typically occurs when the first-phase mSPV exceeds 50°/s [1], which was not seen in our patient. Lastly, short-term VOR adaptation generally requires a marked asymmetry in vestibular input, usually with a high initial nystagmus velocity [3,4,6]. Since none of these hypotheses adequately explain our patient’s findings, the most plausible explanation is the coexistence of canalolithiasis and cupulolithiasis within the right HSC, while detachment of the cupular otoconia explains the disappearance of biphasic nystagmus in the right-ear-down position.

Although the positional nystagmus pattern in this case was atypical, the initial ocular motor and neurological examinations demonstrated no central signs. In addition, the patient’s symptoms resolved after the repositioning maneuver, and therefore neuroimaging was not performed.

Another key takeaway from this case is that, if the affected ear in HSC-BPPV (canalolithiasis) is incorrectly identified, then performing the Lempert roll maneuver on the healthy side may result in conversion to cupulolithiasis. Although spontaneous conversion to cupulolithiasis cannot be completely excluded, it is less likely given the temporal relationship between the maneuver and symptom persistence.

A previous case report described conversion from canalithiasis to cupulolithiasis after the Lempert roll maneuver [20]. In addition, Nuti et al. reported that rapid rotation of the head toward the presumed unaffected side in the supine position could convert geotropic HSC-BPPV to an apogeotropic pattern, suggesting migration of otoconia toward the ampullary side of the canal [21]. The procedure used by Nuti et al. involved rapid rotation of the head alone and was therefore not identical to the Lempert 360° roll maneuver. Nevertheless, the direction of head rotation toward the presumed unaffected side was the same as the direction of rotation used in the present case. Thus, although the two procedures should not be considered equivalent, the previous observation provides a possible mechanical precedent for the positional change observed after the maneuver in our patient. Furthermore, previous reports and mechanical models suggest that the sequence of the supine head-roll test and the initial location of otoconia may influence the observed nystagmus pattern and subsequent lateralization [22].

Although the findings in the present case do not completely conform to these previously proposed mechanical models, they are broadly consistent with the possibility that the sequence of positional testing and the initial location of otoconia may influence the observed nystagmus pattern and lateralization. The change in the positional nystagmus pattern observed on the day after the Lempert roll maneuver raises the possibility that the maneuver, performed on the basis of an incorrect identification of the affected side, may have contributed to the subsequent change in nystagmus pattern. However, the causal relationship cannot be established from this single case, and spontaneous conversion cannot be completely excluded.

Biphasic nystagmus is typically observed when the affected ear is down, making it useful for identifying the affected side [5]. However, the present case demonstrates that biphasic nystagmus can also appear when the healthy ear is down. Thus, before initiating therapeutic maneuvers, clinicians should interpret positional test results carefully and identify the affected side based on comprehensive findings, including the bow and lean test [13]. In a previous report [20], biphasic nystagmus was observed on the side with weaker nystagmus during the head-roll test, which accurately indicated the affected side, and the Lempert roll maneuver was successfully performed. In contrast, in the present case, the affected side was the one where biphasic nystagmus was not observed.

## Conclusions

In conclusion, biphasic nystagmus may occur with the presumed healthy ear-down position during the supine head-roll test. In patients with HSC-BPPV (canalolithiasis), incorrect identification of the affected ear may lead to an inappropriate Lempert 360° roll maneuver and unsuccessful or delayed treatment. In the present case, the change in the positional nystagmus pattern after the maneuver raises the possibility that the maneuver may have contributed to the subsequent development of a positional nystagmus pattern consistent with cupulolithiasis; however, a causal relationship cannot be established from a single case, and spontaneous conversion cannot be excluded. These findings suggest that biphasic nystagmus alone may not reliably identify the affected ear, particularly when other positional nystagmus findings are discordant. The coexistence of canalolithiasis and cupulolithiasis provides one possible pathophysiological explanation for the findings, but this hypothesis requires further investigation.
